# Examination of Marine-Based Cultivation of Three Demosponges for Acquiring Bioactive Marine Natural Products

**DOI:** 10.3390/md9112201

**Published:** 2011-11-07

**Authors:** Oded Bergman, Boaz Mayzel, Matthew A. Anderson, Muki Shpigel, Russell T. Hill, Micha Ilan

**Affiliations:** 1 Department of Zoology, George S. Wise Faculty of Life Sciences, Tel Aviv University, Tel Aviv 69978, Israel; E-Mails: odedbergman.tau@gmail.com (O.B.); bmayzel@gmail.com (B.M.); 2 Institute of Marine and Environmental Technology, University of Maryland Center for Environmental Science, Columbus Center, Suite 236, 701 East Pratt Street, Baltimore, MD 21202, USA; E-Mails: andersomenator@gmail.com (M.A.A.); hill@umces.edu (R.T.H.); 3 National Center for Mariculture, IOLR, P.O. Box 1212, Eilat 88212, Israel; E-Mail: mshpigel@ocean.org.il

**Keywords:** Denaturing Gradient Gel Electrophoresis (DGGE), mariculture, marine natural products, Red Sea, aquaculture

## Abstract

Marine sponges are an extremely rich and important source of natural products. Mariculture is one solution to the so-called “supply problem” that often hampers further studies and development of novel compounds from sponges. We report the extended culture (767 days) at sea in depths of 10 and 20 m of three sponge species: *Negombata magnifica*, *Amphimedon chloros* and *Theonella swinhoei* that produce latrunculin-B, halitoxin and swinholide-A, respectively. Since sponge-associated microorganisms may be the true producers of many of the natural products found in sponges and also be linked to the health of the sponges, we examined the stability of the bacterial communities in cultured *versus* wild sponges. Growth rate of the sponges (ranging from 308 to 61 and −19 (%)(year^−1^) in *N. magnifica*, *A. chloros* and *T. swinhoei*, respectively) differed significantly between species but not between the two depths at which the species were cultivated. Survivorship varied from 96% to 57%. During culture all species maintained the content of the desired natural product. Denaturing gradient gel electrophoresis analysis of the sponge-associated bacterial consortia revealed that differences existed between cultured and wild sponges in *T. swinhoei* and *A. chloros* but the communities remained quite stable in *N. magnifica*. The cultivation technique for production of natural products was found to be most appropriate for *N. magnifica*, while for *T. swinhoei* and *A. chloros* it was less successful, because of poorer growth and survival rates and shifts in their bacterial consortia.

## 1. Introduction

In recent decades, sponges have emerged as one of the most prolific sources for discovery of novel marine-derived secondary metabolites [1,2]. Many of these natural products exhibit a wide variety of biological activities, including anti-cancer, anti-inflammatory, anti-bacterial, anti-fungal, anti-viral, and anti-parasitic activities [3–5]. These discoveries indicate the great potential for biotechnological applications of sponge secondary metabolites, possibly as potential new drugs (or leads). However, concentrations of the desired compound are usually very low [6]. Harvesting sponges from nature is ecologically and economically not a valid option because of low sponge abundance [7]. This is known as the “supply problem” [4]. The most direct solutions for this problem, chemical synthesis or semi-synthesis are frequently either not feasible, or economically non-viable [8]. Culturing sponge cells *in vitro* either as primmorphs [9–11], or cell lineages [12,13], are short term methods [14,15], and not enough is known about factors influencing such culture [8,13]. Thus mass production by these methods has so far not been achieved. Molecular approaches such as isolation of gene clusters involved in biosynthesis have been examined, and could be the solution for future production of some of these desired compounds. However many of the biosynthetic pathways of these sponge metabolites are highly complex and frequently unknown [8]. Another solution that has gained interest in recent decades is the establishment of marine-based mariculture systems (*i.e*., open systems) of explants cut from parental sponges (source individuals) [16].

Culturing sponges is biotechnologically simple, non-expensive to build and maintain, uses the ocean as an almost unlimited bioreactor and relies on the sponge regenerative abilities [16,17]. Semi-closed or closed systems could enable better control over environmental conditions during culture [18], possibly allowing annual rather than seasonal culture (depending on the species) [19] but cultivation in a controlled environment is to date problematic [7]. Proper handling procedures of fragments are vital to minimize damage and allow for fast recovery [20]. To achieve a high yield of a natural product from sponge culture, growth rate, survival, and metabolite production are the most important factors [21]. Sponge growth rate can vary significantly between species reaching a phenomenal annual growth rate of >4000% [22]. Sponge culture has a long history and as Milanese *et al.* [23] state “Natural bath sponges … have been harvested for millennia” and the trade in them flourished. More recently sponge farming was established with newer methods and materials introduced in the last two decades (e.g., [21,22,24–29]) improving sponge survival and growth rate. Most of these sponge cultivation experiments lasted short periods of time (months) and can therefore only be used as estimates for long-term cultivation. Long-term experiments are needed in order to fully understand their effectiveness.

Sponges contain a variety of diverse microorganism communities from various phyla and kingdoms belonging to different domains in marine sponges (*i.e*., bacteria, fungi, microalgae, archaea and protozoa) [30,31]. Bacterial abundance can reach more than 10^9^ cells/mL sponge (up to 30% of the sponge volume), exceeding that of seawater by more than three orders of magnitude [31,32]. Alternatively, these microorganisms may occupy a very small proportion, depending on the sponge species [33]. Some sponges contain high numbers of a single bacterial species, (e.g., *Oscillatoria spongelliae* which comprises about 50% of *Dysidea herbacea* volume) [34]. Sponges that contain numerous bacteria in their mesohyl matrix are termed “bacteriosponges” or “high-microbial-abundance (HMA) sponges” [35]. The bacterial load appears to be correlated with the filtration system. Generally, HMA sponges have a denser mesohyl, a more complex aquiferous system, and thus a slower water flow [30,36]. Sponge-associated bacteria have been suggested to fulfill many functions with regard to their host, including: supply of nutrients, skeletal stabilization, waste products processing, and secondary metabolite production [32], and thus contribute to the sponge’s health and nutrition [33,37]. Utilization of molecular tools enabled greater insight as to the composition of the sponge’s microbial communities, revealing common microbial signatures in many sponges collected at different locations. While phylogenetically highly complex, these communities differ from those in the surrounding water column environment [32,38], although recently deep sequencing revealed that some of the previously considered sponge exclusive bacteria, could be rarely found also in the surrounding seawater [39]. Generally, temporal and biogeographic variations in sponge bacterial communities seem to be relatively low [40], but some exceptions have been noted [31]. Several studies have shown that environmental conditions may affect sponges’ health and their microbial community composition. These include temperature elevation [41], exposure to heavy metals [42], and disease [43]. Denaturing Gradient Gel Electrophoresis (DGGE) analysis found multiple sequences exclusively present in diseased *Aplysina aerophoba* sponges, or alternatively in healthy ones [43]. Surprisingly, the microbiology of aquacultured sponges is poorly understood [31]. Changes in sponge microbial composition, following aquaculture or transplantation, appear to be sponge specific, but conflicting reports exist [44]. Shifts in microbial composition, have been noted for some sponges. Examination of bacterial communities of two sponge species upon transfer to aquaculture in a closed system indicated a significant increase in the diversity of the communities associated with the aquacultured sponges, compared to wild ones [37,45]. Similar findings were reported for *Clathria prolifera*, acclimatized for two months in aquaria and cultured in a flow-through or recirculation system [46]. Stable microbial communities were reported in transplanted *Aplysina* spp. sponges [47,48]. For example, while in a brief experiment (11 days) no significant differences were noted in the microbial community [47], the same species, after cultivation of a few months completely lost this community [49]. The sponge *Rhopaloeides odorabile* was cultured in three conditions: (1) *Ex situ* (natural environment), (2) *In situ* (flow-through aquaria), and (3) *In situ* (large mesocosm systems) [44]. The first two lasted 12 weeks and the third a year. A shift in bacterial community was noticed only in the last condition, indicating the importance of long-term experiments.

**Figure 1:**
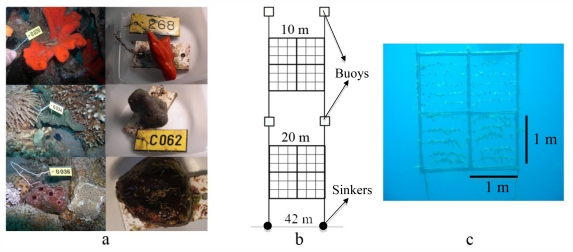
Mariculture system layout. (**a**) Studied sponge species (wild on left, cultured fragments on right) from top: *N. magnifica*, *A. chloros* and *T. swinhoei*; (**b**) Mariculture system design consisting of two plastic frames. The frames were suspended at the desired depth (10 and 20 m) by anchors and buoys. Seabed depth at the culture site was 42 m; (**c**) Each frame consisted of four detachable squares, 1 m^2^ each (see text for further details).

The present long-term study examined the culture amenability of three Red Sea demosponges: *Negombata magnifica*, *Theonella swinhoei*, and *Amphimedon chloros* (Figure 1a), in an open system. These species were chosen because of their metabolite contents having the potential to constitute novel drugs or leads (Table 1). In addition, these species can reach a relatively large size, which makes them suitable for cultivation. Earlier Red Sea surveys found all these species to be common at the study site [26,50–53]. The work also compared between the bacterial communities patterns (by DGGE analysis) of reef sponges, and those from the adjacent mariculture system. Stability of the bacterial communities was checked to assess the sponge health upon transfer to a mariculture facility.

## 2. Results and Discussion

### 2.1. Analysis of Environmental Conditions

Temperatures varied significantly between the two depths (Z_(df5403)_ = −51.607; *P* = 0.001), and were on average (±S.E.) 23.4 ± 0.02 °C at 10 m, and 23.5 ± 0.02 °C at 20 m. This small difference, though statistically significant is due to the large sample size, and probably has less biological significance. Moreover, the S.D. of the temperatures measured at this time period, for each of the two depths is above 1.2 °C. Light intensity differed significantly (Z_(df5403)_ = −37.480; *P* = 0.001) between the depths with means (±S.E.) of 18.3 ± 0.7 lum/ft^2^ at 10 m, and 3.8 ± 0.08 lum/ft^2^ at 20 m.

### 2.2. Growth and Survival

**Figure 2:**
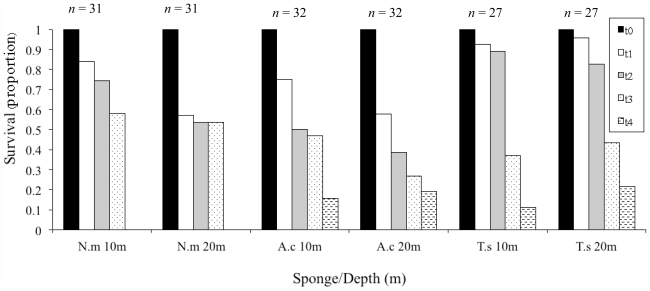
Cumulative survival in proportion for *N. magnifica* (N.m), *A. chloros* (A.c), and *T. swinhoei* (T.s), at 10 m and 20 m. Survival was monitored for the consecutive time intervals (each lasting six months), with t0 representing the beginning of the experiment (t1 = month 1–6, t2 = month 7–12, t3 = month 13–18, t4 = month 19–24).

The survival proportion after the first time interval (first six months) greatly differed between the three cultured species, and to some extent between the two depths (Figure 2). *T. swinhoei* showed the highest survival (93% and 96% at 10 m and 20 m, respectively). Relatively large differences between the two depths were noted for fragments of *N. magnifica* (84% and 57%) and *A. chloros* (75% and 57%). These differences were dramatically reduced to 4% for *N. magnifica* (58% and 54%) by the third time interval and 3% for *A. chloros* (16% and 19%) by the fourth. Surprisingly, *T. swinhoei* survival dropped sharply by the fourth time interval (to 11% and 22%)(Figure 2).

The survival rates reported for *N. magnifica* lie within the range reported in the literature for open system culture of other sponge species (Table 1). In contrast, the survival percentages noted for *A. chloros* and *T. swinhoei* were low compared to other studies. All *N. magnifica* fragments died during the fourth time interval (592 days from the onset of the experiment), as a result of a pathogenic infection. High-density aquaculture may increase the potential for disease outbreaks [60]. However, although the speculation of density-dependent disease infection is tempting, it is still not clear whether a high-density sponge population will increase its vulnerability to disease [61]. Moreover, the three sponge species were co-cultured on the same facility but only *N. magnifica* fragments died. It should be noted that fragment growth was measured a week prior to the disease outbreak. During this processing the fragments were held in the same container until re-attached to the aquaculture installation. In that case it could be that the handling of the fragments in the laboratory promoted the transfer of the infection between all fragments.

**Figure 3:**
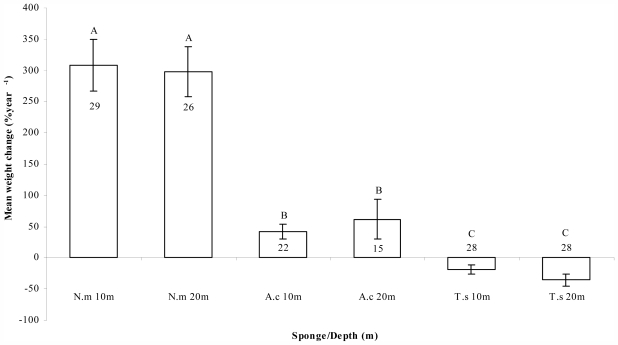
Mean percentage weight change (±S.E.) per year for *N. magnifica* (N.m), *A. chloros* (A.c), and *T. swinhoei* (T.s), at 10 m and 20 m. Growth rate percentage for each individual was determined by comparison of final to initial weight. Number of samples is given in or above the bars. Letters above bars represent statistically significant groups.

Significant differences were noted in final to initial growth rates, as mean weight change (%) year^−1^, for the three cultured sponge species, (F_df(2, 52)_ = 69.214; *P* < 0.001), with no significant difference between the two depths (F_df(1, 52)_ = 1.172; *P* < 0.284) (Figure 3). *N. magnifica* showed the highest growth rates (309 ± 42 at 10 m and 298 ± 40 at 20 m), while fragments of *A. chloros* grew much more moderately (42 ± 12 at 10 m and 61 ± 32 at 20 m) and *T. swinhoei* fragments had negative growth rates (−19 ± 9 at 10 m and −36 ± 10 at 20 m) (Figure 3). In a similar study conducted at the same location [26], growth rates for *N. magnifica* were very similar (328.5 ± 29.2%); however, the experiment lasted only about six months, whereas the current study shows that the growth rates can be sustained over a much longer period of time. Analysis of the growth rates reported in this study, reveal a similar picture to that of the survival rates demonstrated by the different sponges. While *N. magnifica* exhibited high growth rates, those of *A. chloros* and *T. swinhoei* are at the lower side of the reported range (Table 2). A possible explanation for the low survival rates, measured for *T. swinhoei* at the third and forth time intervals, is the loss of weight over time, which may have reached a critical threshold and caused fragment mortality.

The interaction of depth and season and their influence on growth rates have been shown by Duckworth *et al.* [62] for two demosponges in New Zealand, *Polymastia croceus* and *Latrunculia wellingtonensis*. Their study demonstrated that environmental factors can have a strong effect on the cultivation of sponges. Growth and survival were found to be lowest during summer when temperatures were highest (annual range 10–20 °C). In contrast, the temperatures at our study site were relatively warm year-round, slightly varying seasonally (annual range 21–27 °C), and in addition, the sea is calm and clear for most of the year [26]. These relatively constant conditions might account for the fact that no significant differences in SGR were found between the two depths for the three studied species.

### 2.3. Chemical Analysis

NMR analysis demonstrated that the three sponge species continuously produced the metabolites of interest, throughout of the duration of the experiment as demonstrated by characteristic NMR profiles.

Examination by NMR of extracts from *T. swinhoei* samples taken from the maricultured frame and from the natural reef showed they all contained swinholide A, and *A. chloros* extracts had halitoxin, at all the different time intervals. In *N. magnifica* the average amount of Lat-B (percent of dry sponge weight) was 0.91 at 10 m, 0.96 at 20 m for the maricultured sponges and 0.72 for sponges collected from the wild. All samples did not differ significantly in Lat-B content (F_df(5, 74)_ = 1.360; *P* = 0.279). An additional extensive examination of monthly latrunculin content in wild and maricultured *N. magnifica* was carried as part of a study focused on *Negombata* spp. in the Red Sea to be published separately [65].

### 2.4. DGGE Analysis

Maintenance of a sponge in aquaculture needs to take into account the important aspect of the sponge-associated microbial community, as some bacteria may influence the sponge’s health and longevity [33]. Therefore, routine monitoring of this community is a crucial aspect in this field of biotechnology [45].

**Figure 4:**
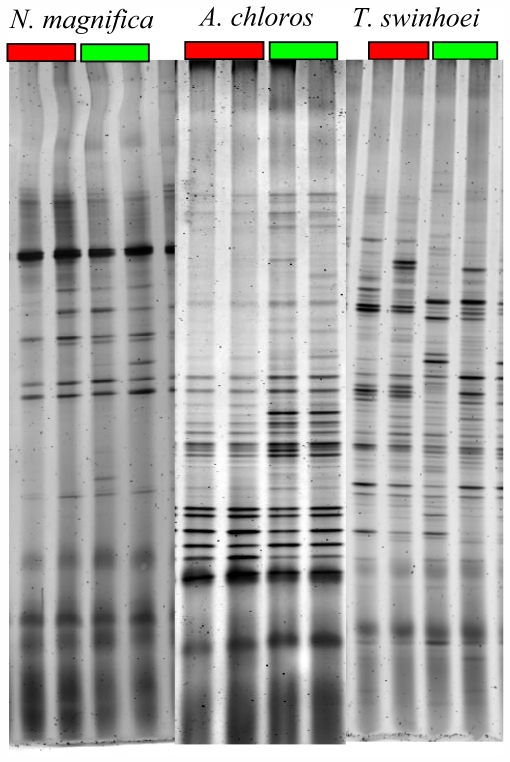
Representative samples of DGGE gels from wild and cultured *N. magnifica*, *A. chloros* and *T. swinhoei*. Each gel contains two samples of wild sponges depicted under the red box and two samples of aquacultured sponges represented under the green box. Samples originating from different sponges were run on separate gels.

**Figure 5:**
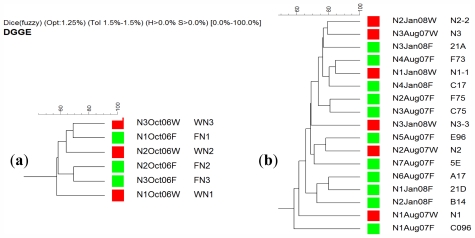
A similarity dendrogram of DGGE banding profiles from *N. magnifica* sponge individuals; panel (**a**) samples collected in 2006 and panel (**b**) samples collected in 2007 and 2008. Each sample’s name represents the individual collected (e.g., N1), time of collection (e.g., October 2006) and whether it was collected from a wild (W) marked in red, or a cultured (F) sponge marked in green.

Analysis of the average number of bands found in gels of sponges collected from the natural reef environment and aquacultured sponges revealed an increase in their number for *A. chloros* (38.1 and 43.1, respectively) indicating an increase in microbial diversity. For the other two species *T. swinhoei* and *N. magnifica*, a decrease was found: (46.9 and 41.9) and (20.7 and 19.7) respectively. An example of a DGGE gel profile is given in Figure 4. This decrease indicates a reduction in bacterial diversity for sponges transferred to an aquaculture facility. The results also indicate that *A. chloros*, and *T. swinhoei* have diverse bacterial communities relative to reported species, with the last being the much more diverse. For example in *Aplysina aerophoba*, between 15–20 bands were present per lane [47], and in five Arctic species between 12–38 bands were resolved per species [66]. However, the number of bands generally found for *N. magnifica* was almost half that found in the profiles of the other two species (Figure 4). For all three sponges, the highest variance was seen between samples that were run on different gels, with two major branches present on the dendrogram each from a different gel. There was no significant difference between the DGGE profiles of wild *N. magnifica* sponges and those from the aquaculture frame setup (Figure 5). Of the three studied species, *N. magnifica* appears to have the most stable bacterial community with little variation based on source or collection date. Many of the branches of the similarity dendrogram contain closely related samples that represent wild and aquaculture sponges, such as N4Aug07F and N1Jan08W (Figure 5b) that are more than 80% similar to each other. Also no variance of DGGE profiles based on collection date exists (e.g., N3Aug07W and N2Jan08W; Figure 5b). These findings correspond with the high growth rates and survival demonstrated by this sponge. Similarly, during three years, the associated bacteria of the Mediterranean *Chondrilla nucula*, studied *in situ*, had high similarities on both the spatial and temporal scales [67].

**Figure 6:**
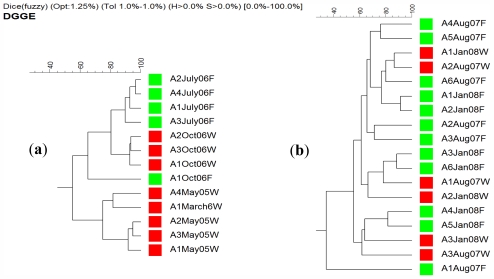
A similarity dendrogram of DGGE banding profiles obtained from *A. chloros* sponge individuals; panel (**a**) samples collected in 2005 and 2006 and panel; (**b**) samples collected in 2007 and 2008. Each sample name represents the individual collected (e.g., A1), time of collection (e.g., May 2005) and whether it was collected from a wild (W) marked in red or a cultured (F) sponge marked in green.

**Figure 7:**
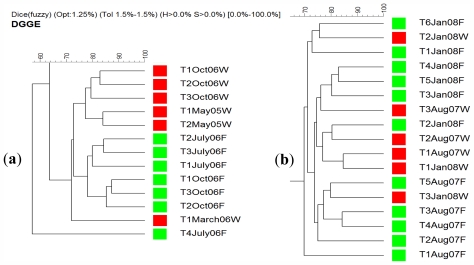
A similarity dendrogram of DGGE banding profiles obtained from *T. swinhoei* sponge individuals; panel (**a**) samples collected in 2005 and 2006 and panel (**b**) samples collected in 2007 and 2008. Each sample’s name represents the individual collected (e.g., T1), time of collection (e.g., May 2005) and whether it was taken from a wild (W) marked in red or a cultured (F) sponge marked in green.

On the other hand *A. chloros* bacterial communities in wild sponges tend to cluster together while those in cultured sponges form their own distinct branches (Figure 6). These results indicate that a change in the bacterial communities occurred when *A. chloros* individuals were placed on an aquaculture frame. A few exceptions to this pattern occurred for some of the individuals, for example a wild sponge A2Aug07W that is most closely related to a cultured one A6Aug07F (Figure 6b), which is a cultured sponge, however this only occurs infrequently. The data demonstrate that time of sampling did not affect the bacterial communities as much as the source (wild *vs.* cultured) of the sponge. For example there is a large branch of closely related wild sponge samples that came from May 2005 and March 2006, and similarly those from October 2006 (Figure 6a) that are completely distinct from any sponges collected from the frame aquaculture setup at the same sampling times. Of the three studied species, *T. swinhoei* sponges had the largest individual variation in their bacterial communities (Figure 4), with many individuals having unique bands only found in that individual. A large amount of variance between samples is based on collection time (Figure 7). Many distinct branches on the similarity dendrogram consist of all individual sponges collected from a single time point. For example a single branched comprised of all samples collected in October 06 (Figure 7a). Interestingly, with a specific collection time there is also variance based on source. For example, all wild sponges from October 2006 clustered together distinctly from a second cluster representing all aquacultured sponges from October 2006. The data suggest that *T. swinhoei* sponges do not have a highly stable bacterial community and that this community changes based both on time of collection and source. *T. swinhoei* also demonstrated a change in the bacterial communities within individuals placed on an aquaculture frame, shown by unique bacterial communities in all sample conditions. The change in microbial community over time, as seen from *T. swinhoei* DGGE analysis, may explain the negative growth rates, and sharp decline in the survival rates in the second part of the experiment. Similarly, during 12 months of *Rhopaloeides odorabile* culture (in a mesocosm system) a change occurred in the microbial community composition over time and loss in sponge biomass [44]. Likewise, *Aplysinella* sp. transplantation experiment, between depths, individuals experienced a substantial loss and gain in volume and demonstrated pronounced changes in the DGGE banding profile [68]. In contrast, transplantation of *Aplysina cavernicola* to shallow water, although resulting in tissue degradation, showed no significant changes in the DGGE banding profiles of sponges from all habitats, indicating relatively unaffected bacterial communities [48].

## 3. Experimental Section

### 3.1. Study Area

Collection of sponge specimens was done at the Israel Oil Terminal (KATZA) pier (longitude 29°31′37, latitude 34°56′04), and at the Interuniversity Institute for Marine Sciences (IUI), (longitude 29°30′07, latitude 34°55′02), both located at the northern tip of the Gulf of Aqaba in Eilat, Israel. All sponges are widely distributed through the study area at depths of up to 20 m. The mariculture system was placed at a distance of about 100 m offshore, adjacent to the IUI pier, where the seabed bottom is at a depth of 42 m. For most of the year the sea is clear and calm, with water temperature fluctuating between 21 °C in winter, to 27 °C in summer [26].

### 3.2. Mariculture System Design

Previously Hadas *et al.* [26] have successfully cultured in the Red Sea fragments of the sponge *Negombata magnifica*, securing and attaching them to PVC plates mounted onto plastic nets. In the current study sponges attached to such PVC plates, were cultivated on a mariculture system, consisting of two plastic frames, each 2 × 2 m^2^ (Figure 1b–c). The two frames were deployed in open water, facing the IUI pier. Both were anchored to the seabed via sinkers and suspended, one at depths of 10 m and the other at 20 m, by floatation barrels. An array of four detachable squares, 1 m^2^ each, was mounted on both frames, using plastic cable ties. The cultivation experiment was initiated in December 2005 and lasted a total of 767 days. Divers cleared the frames of fouling organisms every 2–3 weeks.

### 3.3. Analysis of Environmental Conditions

HOBO Pendant Temperature and Light Data Logger, model UA-002-08 was used for measuring relative light intensity (lum/ft^2^) and temperature. Data-loggers were mounted onto both frames, at 10 m and 20 m. The data was later analyzed using HOBO were pro software (version 2.2.1).

### 3.4. Sponge Collection and Preparation

Specimens of the three sponge species were collected from the reef by using SCUBA at depths of 5–18 m, at the study area. Sponges were harvested from the wild by leaving a part attached to the substratum to allow proper regeneration. Altogether 34 individuals of *N. magnifica*, 38 of *T. swinhoei*, and 41 of *A. chloros* were cut underwater, tagged, and placed in separate seawater filled plastic Ziploc bags. Submerged in seawater, sponges were put in a flow-through seawater system. From each individual sponge eight equal-sized fragments were cut, two for DNA extraction and the remaining six for cultivation on the mariculture facility. For further information see Bergman *et al.* [69].

### 3.5. Annual Specific Growth Rate (SGR) Measurements and Survival Monitoring

Three randomly chosen fragments of each individual sponge were placed on each frame, using a pre-weight PVC plate. Growth rate, determined using the Wet Weight method [70] and calculated as the mean of each individual’s three replicates, and survival measurements were monitored and recorded at six-month intervals. A sponge was considered as surviving to the time of examination if it maintained at least some parts alive. Annual specific growth rates were determined, by comparing the initial wet weight to the final one, using the following equation: $\frac{W_{t}-W_{0}}{W_{0}}\times 100\times D^{-1}\times 365$, where *W*t and *W*_0_ are final and initial weight, respectively, and D is the duration of the experiment in days.

At each time interval, individuals of all sponge species cultured were collected from the two sources: The mariculture system at a depth of 10 m, and wild reef sponges from the adjacent to the IUI center. Each sponge sample was placed on a sterile cutting surface and sampled for microbial cultivation and molecular analysis (by DGGE). A more detailed depiction of the SGR and survival measurements can be found in [69].

### 3.6. Chemical Analysis

A qualitative validation of natural product content was done for latrunculin B (Lat-B) from *N. magnifica*, swinholide A from *T. swinhoei*, and halitoxin from *A. chloros*. At the different time intervals, samples of each sponge were taken for chemical analysis, from the frames (10 m and 20 m) and from the natural reef. Samples were frozen (by liquid nitrogen, and transferred to −80 °C) immediately after collection. Samples were later lyophilized, extracted (dichloromethane:methanol 9:1), and examined by ^1^H-NMR 400 MHz. Compounds present in the extracts were determined based on the following: Latrunculins-Both latrunculin A and B possess two characteristic methyls, a doublet at 1.0 and a singlet at 1.95. In addition latrunculin A possesses five vinyl protons the lowest one a double doublet at 6.4, while latrunculin B possesses only three vinyl protons the lowest one at 5.25 t. Swinholide A-7.26 d and 5.35 d (*J* = 16 Hz), 3.18 s and 3.21 d—two OMe groups, 1.70 brs a vinyl methyl, as well as four additional sp^3^ methyl doublets. Halitoxin—8.90, 8.81, 8.44 and 8.00 (one proton each)—four pyridinium protons, as well as long aliphatic chains.

Twenty-three sponge fragments of *N. magnifica* were sampled, weighted, frozen using liquid nitrogen, and transferred to −80 °C. The samples were later lyophilized and weighted using an electronic balance (to 0.1 g). The fragments were cut into small pieces, soaked in dichloromethane (DCM) and placed on a shaker, for 24 h at 75 rpm. The obtained crude extract was filtered through a filter paper (Whatman 595 ½ folded filters Ø150 mm) and evaporated using a vacuum rotary evaporator (BÜCHI, Rotavapor R-114), until completely dried. The process was repeated twice for 24 h and a third time for six hours. The final dried crude extract was dissolved in methanol at 5 mg/mL and diluted to 1 mg/mL. Of each sample 20 μg were injected into the HPLC (AJILENT 1100), equipped with G1315B DAD Diode Array detector, set on 225 nm and 30 °C and a Phenomenex Luna 5 μ, type C-18 (2) column. The initial medium used for the separation was water:methanol (1:1). Rate of flow was 1 mL/min and injection time 41 min/sample. The time and gradient of the medium used were: 0–30 min 50% methanol up to 100%, 30–35 min 100% methanol, 35–41 min 50% methanol. Detection time for latrunculin A was 23 min and for latrunculin B 25.6 min (standards kindly provided by Y. Kashman Tel Aviv University School of Chemistry). The peak area obtained was analyzed using the HPcame.

### 3.7. Denaturing Gradient Gel Electrophoresis (DGGE)

For DGGE analyses a total of 23 *N. magnifica* individuals were collected over three time points spanning one and a half years (between October 2006 and January 2008). Nine individuals were collected from the surrounding reef and 14 from the open water frame aquaculture setup. A total of 31 *A. chloros*, and 30 *T. swinhoei* individuals were collected over six time points spanning almost three years (between May 2005 and January 2008). Fourteen *A. chloros* individuals were collected from the surrounding reef and 17 from the open water frames, while 12 and 18 (respectively) *T. swinhoei* individuals were collected. Samples of each sponge were processed separately by DGGE on two different gels because of their large number. The gels were analyzed separately because of the difficulty of accurate comparison between gels. The study, nonetheless, used DGGE as a tool for intra gel comparison. Samples comparing wild *vs*. aquacultured sponges for a given year were always run together, with the same PCR reaction and DGGE gel. Therefore, a low-resolution, or existence of co-migration based on gradient imperfections, should be comparatively the same for those samples. Although the resolution of DGGE imposes some limitations on detection of the entire diversity of bacteria present in complex communities, comparisons of the diversity as represented by number of DGGE bands should hold valid within the context of a gel’s samples.

Diversity of bacterial communities within sponge fragments was analyzed by DGGE of 16S rRNA gene fragments. A small section of each sponge individual (1 cm^3^) was lyophilized for DNA extraction. Lyophilized tissue was ground to powder using a sterile mortar and pestle. For DNA extraction, 100 mg of the powdered tissue was placed in a 2 mL microcentrifuge tube. Sponge tissue was covered with 1 mL of 1× TE buffer, 500 L of guanidine thiocyanate buffer, and a metal Tissuelyzer ball was added. The tube was placed in a Tissuelyzer (Qiagen, Valencia, CA, USA) and shaken at 220 rpm for 2 min. This homogenate was put on ice and ammonium acetate was added to 2.5 M. The homogenate was cleaned up using a phenol:chloroform (1:1) method coupled with isopropanol precipitation for DNA isolation. Resulting DNA was re-suspended in 1× TE buffer. This DNA was used for DGGE analysis. A 200 bp fragment of the 16S rRNA gene was PCR-amplified from total community DNA using the P2 (ATTACCGCGGCTGCTGG) and P3 (CGCCCGCCGCGCGCGGCGGGCGGGGCGGGGGCACGGGGGGCCTACGGGAGGCAGCAG) primers [71]. The following PCR conditions were used: 95 °C for 5 min, 94 °C for 1 min, 55 °C for 1 min, 72 °C for 1 min (steps 2–4 for 30 cycles), 72 °C for 5 min. DGGE was performed using a Bio-Rad DCode system (Bio-Rad, Hercules, CA, USA) with an 8% (wt/vol) polyacrylamide gel and a denaturing gradient of 40%–75% in 1× Tris-acetate-EDTA buffer. The gel was run at 60 V for 18 h at 60 °C. Gels were stained with SYBR gold (20 mL SYBR gold/500 mL 1× TAE) for 30 min and imaged with a Typhoon 9410 image system (Amersham Biosciences, Piscataway, NJ, USA).

GelComparII software (Applied Maths, Kortrijk, Belgium) was used to analyze DGGE fingerprint profiles. Images of DGGE gels were imported into the program and similarity dendrograms were constructed using the Dice Coefficient (2% tolerance, 1.25% optimization) with a UPGMA calculation. Density of bands was not considered in this analysis.

### 3.8. Statistical Analysis

The SGR data obtained from *N. magnifica*, *A. chloros*, and *T. swinhoei* individuals at both 10 m and 20 m was analyzed by repeated measure analysis of variance (MANOVA) with the variables Species and Depth. To normalize the data log transformation was used. Differences in Lat-B content within *N. magnifica* were analyzed by a one-way ANOVA.

## 4. Conclusions

In this long-tern study, marine based aquaculture system has been designed for the accumulation of biomass and the extraction of secondary metabolites as one solution to the supply problem. Growth rates, survival, production of the metabolites of interest and sponge health were routinely monitored. The culture technique was found to be most appropriate for the sponge *N. magnifica*. With a growth rate in excess of 300% per year, and high survival rates, this sponge is a good candidate for large-scale mariculture experiments. Moreover, the growth rate monitored for this sponge increased over time and the metabolite of interest, Lat-B, was constantly produced at similar concentrations of the wild population, even after 18 months of culture. DDGE analysis revealed a stable bacterial community in this sponge, with little variation based on source or date of collection, indicating the health state of cultured sponges was good. The mariculture of *T. swinhoei* and *A. chloros* was not successful, yielding poor growth and survival rates. Concurrently the DGGE analysis of both species showed an unstable bacterial community, varying between habitat (wild and aquaculture) and season of sample, possibly suggestive of sponges in poor health. These latter results render the two species as non-suitable for aquaculture in the described system. Thus the decision whether to culture a sponge for production of its secondary metabolites should be made based on experience with the species of interest and the culture system, since no general rule applies for all species in the various culture methods.

## Figures and Tables

**Table 1:** Main secondary metabolite and bioactivity of the studied sponge species.

Species	*T. swinhoei*	*N. magnifica*	*A. chloros*
Order and family	“Lithistida” Theonellidae	Poecilosclerida Podospongiidae	Haplosclerida Niphatidae
Main secondary metabolite	Swinholide A (Polyketides) [54]	Latrunculin B (Polyketide) [55]	Halitoxin (Pyridinium alkaloids) [56,57]
Bioactivity	Cytoskeleton affector: Antiactine Anticancer [59]	Anticancer [58]	Antimicrobial [57]

**Table 2:** Maximal specific growth rate of sponges cultured in an open system.

Sponge species	Variable measured	Maximal Growth rate * (%)(year^−1^)	Survival (%)	Duration of experiment	Reference
*Mycale cecilia*	Volume	1260	95	60 days	[7]
*Latrunculia wellingtonensis*	Weight	675	56	285 days	[21]
*Geodia cydonium*	Weight	378	-	3–6 months	[63]
*Agelas oroides*	Volume	336	-	15 months	[64]
*Polymastia croceus*	Weight	260	59	285 days	[21]
*Spongia officinalis*	Wet weight	120	75	3 years	[25]
*Haliclona oculata*	Volume	4.4	-	1 year	[27]
*Negombata magnifica*	Wet weight	324	Between 40 and 75	177 days	[26]
*Negombata magnifica*	Wet weight	308	** 54	592 days	Current study
*Amphimedon chloros*	Wet weight	61	19	767 days	Current study
*Theonella swinhoei*	Wet weight	−19	11	767 days	Current study

* Increase in maximal growth rate calculated as the change in the initial to final weight (or volume). When different culture methods were used, only the maximal result is presented (and the corresponding survival percentage, if available);

** Survival of the fourth time interval was omitted, as a pathogenic infection caused a mortality of all the cultured stock.
